# Viral escape mutations do not account for non-protection from SIVmac239 challenge in RhCMV/SIV vaccinated rhesus macaques

**DOI:** 10.3389/fimmu.2024.1444621

**Published:** 2024-08-07

**Authors:** Benjamin N. Bimber, Justine Sunshine, G. W. McElfresh, Jason S. Reed, Reese Pathak, Katherine B. Bateman, Colette M. Hughes, Roxanne M. Gilbride, Julia C. Ford, David Morrow, Jeffrey D. Lifson, Jonah B. Sacha, Scott G. Hansen, Louis J. Picker

**Affiliations:** ^1^ Oregon National Primate Research Center, Oregon Health and Science University, Beaverton, OR, United States; ^2^ Vaccine and Gene Therapy Institute, Oregon Health and Science University, Beaverton, OR, United States; ^3^ AIDS and Cancer Virus Program, Frederick National Laboratory, Frederick, MD, United States

**Keywords:** SIV, viral escape, CMV vaccine vector, viral sequence analysis, HIV/SIV

## Abstract

Simian immunodeficiency virus (SIV) vaccines based upon 68-1 Rhesus Cytomegalovirus (RhCMV) vectors show remarkable protection against pathogenic SIVmac239 challenge. Across multiple independent rhesus macaque (RM) challenge studies, nearly 60% of vaccinated RM show early, complete arrest of SIVmac239 replication after effective challenge, whereas the remainder show progressive infection similar to controls. Here, we performed viral sequencing to determine whether the failure to control viral replication in non-protected RMs is associated with the acquisition of viral escape mutations. While low level viral mutations accumulated in all animals by 28 days-post-challenge, which is after the establishment of viral control in protected animals, the dominant circulating virus in virtually all unprotected RMs was nearly identical to the challenge stock, and there was no difference in mutation patterns between this cohort and unvaccinated controls. These data definitively demonstrate that viral mutation does not explain lack of viral control in RMs not protected by RhCMV/SIV vaccination. We further demonstrate that during chronic infection RhCMV/SIV vaccinated RMs do not acquire escape mutation in epitopes targeted by RhCMV/SIV, but instead display mutation in canonical MHC-Ia epitopes similar to unvaccinated RMs. This suggests that after the initial failure of viral control, unconventional T cell responses induced by 68-1 RhCMV/SIV vaccination do not exert strong selective pressure on systemically replicating SIV.

## Introduction

Cytomegalovirus (CMV) was originally pursued as a vaccine vector for HIV based on the concept that the unique immune adaptations of this persistent β-herpesvirus would result in robust, widely distributed effector-memory (EM) T cell immunity against encoded SIV inserts, which would provide an earlier and therefore more effective CD8^+^ T cell-mediated interception of nascent HIV/SIV infection in portals of viral entry and sites of early viral spread. Consistent with this hypothesis, RM vaccinated with SIV insert encoding vectors based on strain 68-1 RhCMV manifested a unique early, all-or-none pattern of stringent viral control after repeated limiting dose, mucosal challenge with highly pathogenic SIVmac239. Over multiple studies, 59% of vaccinated RM stringently control SIV replication in the first 7-14 days following effective challenge, either remaining aviremic or manifesting only transient plasma viral blips (1). Initially, protected monkeys are demonstrably SIV infected as shown by the presence of viral RNA and DNA in tissues, development of *de novo* T cell responses to SIV antigens not in the vaccine, and by demonstration of replication competent and fully pathogenic virus in tissues by adoptive transfer; however, over ensuing months to 1-2 years, both virologic and immunologic evidence of SIV infection wane to extinction, consistent with infection clearance (2–4). This remarkable outcome is thought to be due to a complete replication arrest of the incoming virus prior to establishment of a long-lived SIV reservoir, with the exhaustion of infection reflecting time-dependent degradation of the relatively short-lived SIV reservoir that existed at the onset of replication arrest (1, 5).

This unique “control and clear” protection against pathogenic SIV challenge is not a general property of cytomegalovirus vectors, but specific to strain 68-1 RhCMV or derivatives and homologues of this lab-adapted strain that acquired a specific set of genetic changes during long-term cell culture (6). Unlike WT RhCMV or conventional viral vaccine vectors, 68-1 RhCMVs exclusively elicit CD8^+^ T cell responses with unconventional Major Histocompatibility Complex (MHC) restriction, recognizing peptides presented by MHC-Ib (MHC-E) or MHC-II molecules rather than conventional MHC-Ia (6–8). These MHC-E and MHC-II-restricted CD8^+^ T cell responses are unusually broad, with an average of 4 epitopes recognized per 100 amino acids of SIV insert antigen and include recognition of both universal epitopes called supertopes (e.g., present in all vaccinated RMs) and RM-to-RM variable epitopes called subtopes. Importantly, replication arrest has been shown to require MHC-E-restricted CD8^+^ T cell responses, as it has proven possible to genetically manipulate RhCMV to generate SIV vectors that exclusively elicit MHC-Ia-, MHC-II-, or MHC-E-restricted CD8^+^ T cell responses (while maintaining similarly robust, EM-differentiated, and durable responses) and only those vectors programmed for MHC-E-restricted epitope targeting were shown to be efficacious (6, 9–11).

While MHC-E-restricted CD8^+^ T cells responses are required, and protection in 68-1 RhCMV/SIV vector vaccinated RMs has been associated with a persistent vaccine-induced innate immune signature featuring IL-15 signaling (12), a detailed understanding of the mechanistic basis of protection vs. non-protection among 68-1 RhCMV/SIV vaccinated RMs has been elusive. Multiple lines of evidence demonstrate that virus in protected RMs is fully replication-competent, including adoptive transfer experiments that transfer cells from protected RMs shortly after the onset of viral control to naïve recipients, resulting in unequivocal establishment of typical SIV infection (4). Since rapid sequence evolution is a hallmark of HIV/SIV infection, and immune-driven selection is a major driver of within-host viral evolution (13, 14), it is possible that non-protection in the setting of 68-1 RhCMV/SIV (which elicits MHC-E-restricted CD8^+^ T cells) or in RhCMV/SIV programmed to elicit MHC-Ia-restricted CD8s is due to viral escape mutations. In this study, we performed deep sequencing of plasma virus in non-protected RhCMV/SIV vaccinated RMs to determine whether viral escape mutation explains lack of protection.

## Materials and methods

### SIVmac239 deep sequencing

Viral sequencing and analysis were adapted from previously published genome wide SIVmac239 sequencing protocols (15). Viral RNA was isolated from virus stocks and plasma samples using QIAamp MinElute Virus Spin Kit following manufacturer’s instructions. Complementary DNA was generated with the SuperScript III One-Step RT-PCR with Platinum Taq (ThermoFisher). We used the following four primer pairs to amplify the SIVmac239 genome with four distinct overlapping amplicons: 5’-TCTTTTATCCAGGAAGGGGTAAT-3’ and 5’-GAGATGTTTGGTTTTTATACCTGGA-3’, 5’-AAAATTGAAGCAGTGGCCATTAT-3’ and 5’-TACTTATGAGCTCTCGGGAACCT-3’, 5’-GGCATAGCCTCATAAAATATCTG-3’ and 5’ATTGCAGAACCTGCCGTTG-3’, and 5’-AGGTGGTGGTCTCTTCATGC-3’ and 5’-ACAGAGCGAAATGCAGTGATATT-3’. Independent RT-PCR reactions were performed on Eppendorf Mastercycler Pro S Thermal Cyclers using the following thermal conditions: 50°C for 30 min; 94°C for 2 min; [94°C for 15 sec, 58°C for 1 min, 72°C for 4 min] x 45 cycles; 68°C for 5 min; and hold at 4°C. The resulting amplicons were purified on a 1% agarose gel and cleaned up using NucleoSpin Gel and PCR Clean-up Kit (Macherey-Nagel). Dual-indexed Illumina MiSeq-compatible libraries were then prepared using the Nextera XT DNA Sample Prep Kit following the manufacturer’s instructions and purified with AMPure XP magnetic beads (Beckman Coulter). Libraries were analyzed on an Agilent 2100 Bioanalyzer using the HS DNA kit (Agilent), normalized to 2 nM, pooled at an equimolar ratio, and sequenced in parallel on an Illumina MiSeq sequencer. Sequence reads were processed as previously described (32, 33). Briefly, raw data were trimmed using Trimmomatic, aligned to the SIVmac239 reference sequence (GenBank Accession No. M33262) using BWA-mem (16–18). All bases of the alignment were evaluated, and single nucleotide polymorphisms (SNPs) and deletion/insertion polymorphisms were called for bases with a PHRED-scaled quality score above 17. Importantly, the identity of the associated read was retained for each SNP, which allows the phase of SNPs to be considered. This information allowed amino acid translations to be calculated based on the sequence of each individual read, thereby correctly translating linked SNPs, as opposed to using the sample consensus sequence. SNP analysis and visualization of mutations was performed using the DISCVR/SequenceAnalysis module (19, 20). Raw sequence data are available in the NIH Short Read Database (SRA) under BioProject PRJNA640430.

### Statistical methods

**Figure 1:**
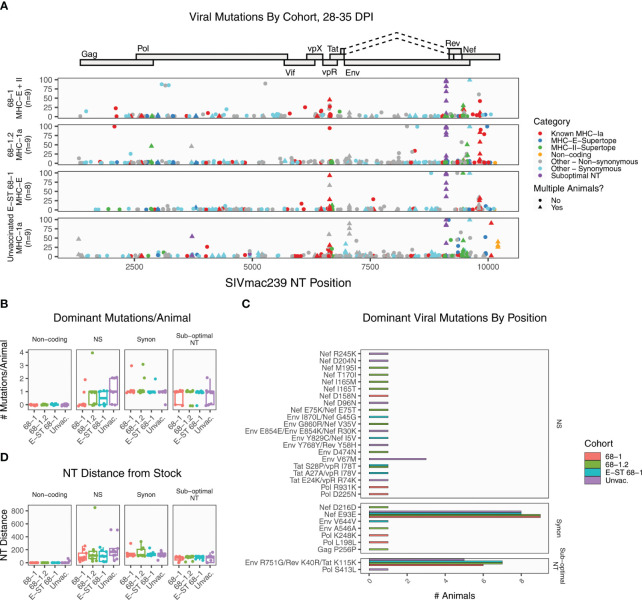
Summary of SIVmac239 viral evolution at 28-35 days post infection. Deep sequencing was performed on plasma virus sampled at 28-35 DPI from four cohorts, identifying the position and frequency of nucleotide mutations. **(A)** The plot displays all detected SIVmac239 mutations in the labeled vaccine cohorts. Each dot represents a single mutation detected in one RM, and the y-axis indicates the frequency of that mutation in the sample. Mutations are colored according to functional category (see legend). **(B)** The graph summarizes the number of dominant mutations (detected in >50% of reads) per animal, grouped by functional category. **(C)** The plot displays every position where a dominant variant was detected in at least one animal, displaying the RMs per cohort with each mutation. Most mutations are private, detected in a single animal. The only shared non-synonymous mutation was Env V67M, detected in three unvaccinated RMs. **(D)** The plot displays the total nucleotide distance from the challenge sequence, defined as the sum of the frequencies of all mutations detected in that sample. Collectively, these data demonstrate that across all vaccine groups, except for known sub-optimal nucleotide A9110G, the dominant circulating virus has nearly identical coding potential to the challenge stock, and there are no significant differences in levels of mutation between cohorts. NS, non-synonymous; Synon, synonymous.


We generated multiple summary statistics to contrast the SIV population between subjects and cohorts. We calculated the total number of dominant mutations (defined as a mutation present in >50% of the viral population) per sample. Additionally, we calculated the total nucleotide distance from the stock in each sample, defined as the sum of the frequencies of all variants in that sample. To contrast mutations between cohorts, we stratified mutations by class (i.e. synonymous, non-synonymous, non-coding, and sub-optimal nucleotides (21)), and performed grouped pairwise Wilcoxon rank sum test (Holm-corrected) to compare mutation rates per cohort, within each class of mutations. An adjusted p value threshold of 0.05 was used in all cases. Analogous pairwise Wilcoxon rank sum tests were also performed to evaluate differences in the rates of mutation within T cell epitopes. We used a Bayesian binomial model to estimate a credible interval for the probability of observing a subject with more than zero dominant non-synonymous mutation within 28-35 days post challenge (**Figure 1B**) based upon the data and sample size of this study (**Supplementary Figure 1**). Model fitting was performed in brms (22), under the following specifications:

Y∼Binomial(N,p)

N=9

p∼TruncNormal(0,1,0,1)

Where TruncNormal is the truncated normal distribution, with mean 0, standard deviation 1, lower bound 0, and upper bound 1. Credible intervals are 80% and 95% highest posterior density interval.

## Results

To test the hypothesis that viral escape explains lack of RhCMV-mediated protection, we longitudinally sequenced plasma SIV from non-protected RM vaccinated with both efficacious and non-efficacious RhCMV/SIV vaccines, including RMs vaccinated with 68-1 RhCMV/SIV vectors (“68-1”, n=9), MHC-Ia-programmed RhCMV/SIV vectors (strain “68-1.2”, n=9), and MHC-E-only 68-1 RhCMV/SIV vectors (miR-126-restricted) encoding E-supertope-enriched SIV inserts (“E-ST-Only”; n=8), all compared to unvaccinated controls (n=9) (7, 9). Importantly, while 68-1.2 elicits MHC-Ia-restricted CD8^+^ T cell responses, these do not target canonical immunodominant epitopes (7). As shown in **Supplementary Figure 2** and **Supplementary Table 1**, these vaccinated RM were all non-protected after challenge, with viral load profiles that closely resembled those of unvaccinated control RM subjected in parallel to the same challenge protocol. Additional details on the RhCMV vectors and cohorts are available in **Supplementary Table 2** and are summarized in a recent review article (1).

To assess SIV sequence evolution, and the potential contribution of immune escape to vaccine non-protection, we performed deep sequencing of plasma viral RNA using established protocols capable of identifying variants and quantifying their intra-host frequency in the viral population (15, 23), initially analyzing plasma viral sequence sampled at 28-35 days post-infection. We selected this timepoint because the vast majority of protected RMs demonstrate SIV replication arrest (e.g., control plasma viremia to below the limit of detection) in the first 7-14 days after effective challenge (2, 3, 5). Thus, if failure of protection is due to viral escape mutations, these mutations should be present in the plasma virus of unprotected RMs at this timepoint, at a sufficient frequency to account for failure to control viral replication. For each RM, we summarized the location and frequency of mutations, and categorized mutations based on coding potential (**Figure 1** and **Supplementary Table 3**). We further categorized variants based on whether the nucleotide position overlaps common T cell epitopes, including conventionally restricted epitopes and MHC-E or MHC-II restricted supertopes. For MHC-Ia-restricted epitopes, a variant is scored as overlapping an epitope only when that RM encodes the restricting MHC-Ia allele for that epitope. It should be noted that while this will identify mutations in common immunodominant epitopes (listed in **Supplementary Table 4**), some mutations could overlap uncharacterized MHC epitopes and are therefore undercounted. Also, the overlap between a variant and epitope does not necessarily indicate that mutation was selected by T cell pressure. Finally, SIVmac239 is a molecular clone selected after *in vitro* passage, and there are four so called “sub-optimal” NT positions previously documented to mutate in virtually all challenged RMs (21). We annotated any variants overlapping these positions.

At this early timepoint, there is a large amount of low frequency mutation in all RMs, as expected; however, there are relatively few dominant mutations (present in >50% of the viral population) in any RM (**Figure 1A**). We detected a total of 37 dominant mutations in total, representing 30 unique positions (**Figure 1B**). The most common mutation across all RMs was sub-dominant nucleotide A9110G, which causes non-synonymous amino acid changes in overlapping reading frames of Env and Rev, with a synonymous change in Tat (**Figure 1A**, purple dots). The emergence of this variant in all RMs indicates that positive viral selection is occurring in all cohorts. Lower frequency mutations were also detected in a second sub-optimal nucleotide: C3721T. Of the remaining dominant mutations, ten (27%) were synonymous variants. Within the 68-1 RhCMV/SIV vaccinated cohort, seven of nine RMs had no non-synonymous changes outside of sub-optimal NT A9110G (**Figure 1B**). The remaining two 68-1 RhCMV/SIV RMs had one and two non-synonymous changes, each. Thus, the dominant circulating virus in this cohort has virtually identical coding potential to the challenge stock. The largest number of dominant mutations per animal occurred in a 68-1.2 RhCMV/SIV vaccinated RM, with four dominant non-synonymous mutations. RMs vaccinated with 68-1.2 RhCMV/SIV generate conventional MHC-Ia restricted CD8^+^ T cell responses, although the epitopes targeted by these responses are non-canonical (typically subdominant epitopes (7)). All four of these changes overlapped with characterized canonical Mamu-A1*002 restricted epitopes, suggesting that if these mutations were CD8^+^ T cell driven, they were driven by *de novo* responses to the SIV infection itself. To determine if there were regions of increased variation between the groups, we then compared the positions of all dominant mutations (**Figure 1C**). As noted, the most frequent site of mutation was sub-optimal NT A9110G. We additionally identified a synonymous mutation (Nef E93E) shared in virtually all RMs. Outside of these positions, virtually all mutations were private, only detected in a single RM. There was a strong skewing of mutations toward the 3’ end of the genome, which is consistent with greater tolerance to variation in those proteins (14, 24). Finally, we calculated the total NT distance from the stock in each sample, defined as the sum of the frequencies of all variants in that sample (**Figure 1D**). These data demonstrate that the dominant circulating virus in unprotected 68-1 RhCMV/SIV vaccinated RMs is nearly identical to the challenge stock in most RMs, and there is no significant difference in the rate or pattern of dominant mutations between vaccinated cohorts and unvaccinated controls. Thus, it is unlikely that escape mutations to vaccine-generated CD8^+^ T cell responses explain the failure of vaccine-mediated control in these animals.

**Figure 2:**
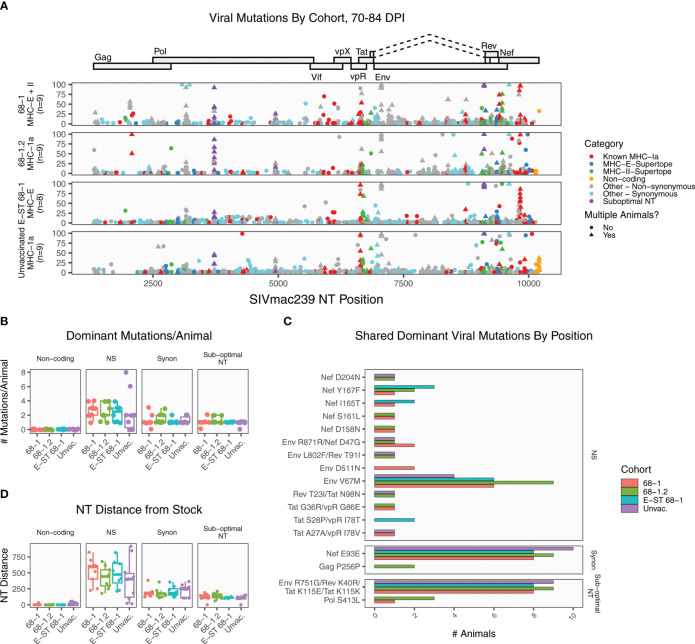
Summary of SIVmac239 viral evolution at 70-84 days post infection. Deep sequencing was performed on plasma virus sampled at 70-84 days post-infection from all cohorts, identifying the position and frequency of nucleotide mutations. **(A)** The plot displays all detected SIVmac239 mutations in the labeled vaccine cohorts. Each dot represents a single mutation detected in one RM, and the y-axis indicates the frequency of that mutation in the sample. Mutations are colored according to functional category (see legend). **(B)** The graph summarizes the number of dominant (detected in >50% of reads) mutations per animal, grouped by category. These data show that all cohorts are accumulating additional dominant mutations, although there are no significant differences between cohorts. **(C)** The plot displays the 17 dominant mutations detected in multiple RMs (out of 58 total dominant mutations), for the purpose of identifying patterns of common selection. While three positions were enriched in every group (Env V67M, Nef E93E, and the sub-optimal nucleotide A9110G), the pattern of mutation was otherwise diverse, and there were not any variants enriched in a specific vaccine cohort. **(D)** The plot displays the total nucleotide distance from the challenge sequence, defined as the sum of the frequencies of all mutations detected in that sample. NS, non-synonymous; Synon, synonymous.

We next performed similar deep sequencing and analysis of viral sequence at 70-84 days post infection. This timepoint, which is two months after immune failure in RhCMV-vaccinated unprotected RMs, provides an opportunity to examine the patterns of viral evolution after immune failure. As expected, all samples accumulated greater levels of mutation relative to the earlier timepoint (**Figure 2A**). Mutation in sub-dominant nucleotide A9110G approached fixation (>90% of reads) in 33 of 35 RMs. The average number of dominant non-synonymous mutations per RM was not significantly different (adjusted p value threshold 0.05, pairwise Wilcoxon rank sum test with Holm corrected multiplicity adjustment) between any of the cohorts at this timepoint (**Figure 2B**). Of the 58 unique dominant amino acid mutations detected, 70.6% were private, unique to a single RM. Three mutations were enriched in every group (Env V67M, Nef E93E, and the sub-optimal nucleotide A9110G). Of the remaining 14 amino acid mutations, most were detected in just two RMs, and none were enriched in a specific cohort (**Figure 2C**). Finally, we calculated the total NT distance from the stock (**Figure 2D**). Collectively, these demonstrate that while a handful of positions are under uniform selective pressure across vaccinated and unvaccinated RMs, most selection is unique per RM.

**Figure 3:**
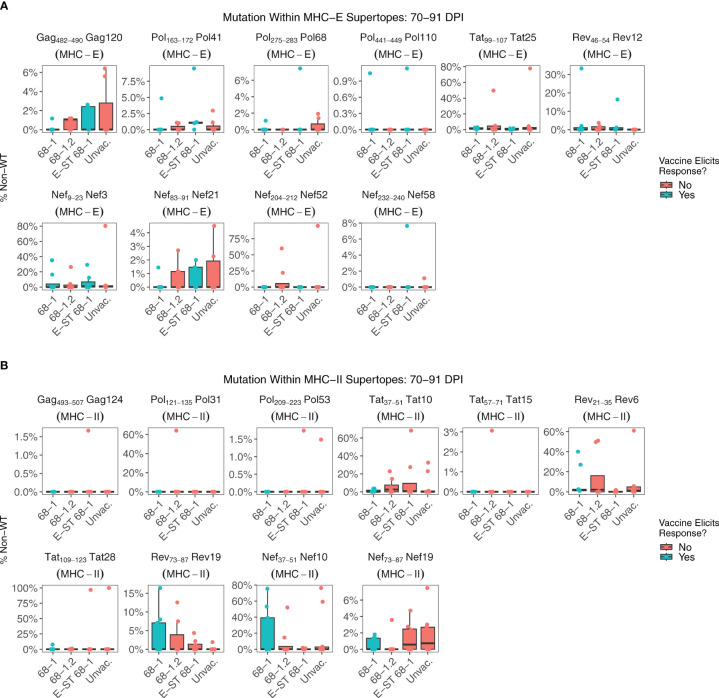
Nucleotide mutation within MHC-E and MHC-II restricted supertopes. Supertopes are a unique property of 68-1 RhCMV vaccines, with different RhCMV versions eliciting different patterns of responses. **(A)** Each boxplot displays the sum of nucleotide mutation per RM within the indicated MHC-E restricted supertope. Plots are colored based on whether the vaccine is expected to elicit a T cell response against that supertope, which is determined by the vector backbone and whether the epitope is encoded by the vector. **(B)** Analogous to **(A)**, each boxplot displays the sum of nucleotide mutation per RM within the indicated MHC-II restricted supertope. Collectively, these data demonstrate that there is relatively little viral mutation within most supertopes of 68-1 RhCMV vaccinated RMs. While there are examples specific RMs with high frequency mutations overlapping a particular supertope, there is no significant difference between RMs that do or do not have a vaccine-elicited response against the supertope, suggesting other factors are driving this selection.

MHC-E restricted CD8^+^ T cell responses are required for 68-1 RhCMV/SIV mediated protection (6, 9–11). All RMs vaccinated with 68-1 RhCMV/SIV vectors generate MHC-E-restricted CD8^+^ T cell responses against a common set of epitopes, termed “supertopes” (8, 9). Because the cytotoxic T cell response is a major driver of intra-host HIV/SIV evolution, shared patterns of viral evolution might be expected in these regions, specifically in RMs that received RhCMV 68-1/SIV or RhCMV 68-1 E-ST vaccines. Each supertope can be categorized based on whether the vaccine vector is expected to elicit an immune response against that epitope and therefore potentially exert selective pressure (**Figure 3**, colors). If there is 68-1 RhCMV/SIV driven immune selection, mutation rate in a given epitope should be greater than in RMs without vaccine-elicited immunity against that epitope. In the vast majority of MHC-E and MHC-II restricted supertopes, little viral mutation was detected in any group (**Figures 3A, B**). Even when higher rates of mutation are observed, rates of mutation are either comparable between vaccinated and unvaccinated cohorts (including the MHC-E- restricted epitopes and Nef 21, and the MHC-II-restricted epitopes Rev 6 and Nef 19), or significant mutation is observed in unvaccinated RMs alone (MHC-E-restricted epitopes Tat 25, and Nef 52, and the MHC-II-restricted epitopes Pol 31, Tat 10, Tat 28). The presence of a viral mutation overlapping a supertope does not necessarily indicate the mutation is driven by T cell selection, and it is likely the patterns of mutation observed in unvaccinated RMs represent either overlapping conventionally restricted T cell epitopes or another source of selection. Thus, while previous studies have shown that unconventionally restricted T cells are essential for the clearance of SIV during the first two weeks of infection, our data show that in RM not protected by vaccination, once infection is established, there is no evidence of widespread vaccine-mediated selection at these sites.

**Figure 4:**
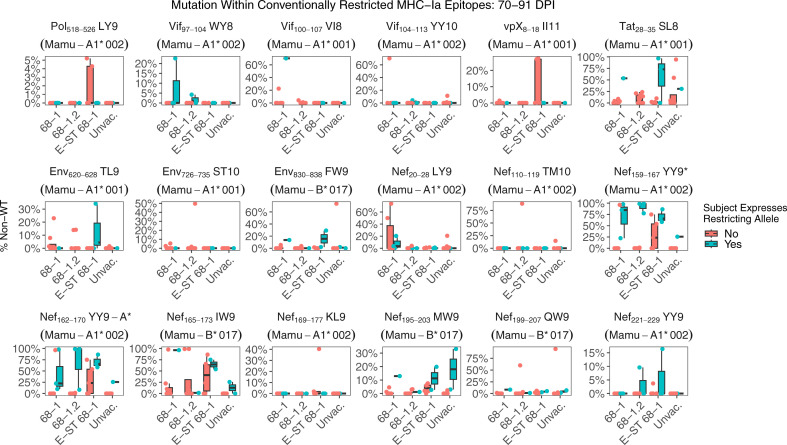
Nucleotide mutation within conventional MHC-Ia restricted supertopes. Each boxplot displays the sum of nucleotide mutation per RM within the indicated immunodominant MHC-Ia restricted epitope. While none of the vaccines elicit responses against canonical MHC-Ia epitopes, *de novo* responses will be primed by the challenge itself. RMs within each group are separated based on whether the RM expresses the restricting MHC-Ia allele for that epitope. Collectively, these data demonstrate that viral escape consistent with MHC-Ia restricted CTL pressure occurs in both RhCMV/SIV vaccinated and unvaccinated RMs. Asterisks after the plot title indicate epitopes with a statistically significant difference in mutation rates between RMs that do or not express the restricting MHC-Ia allele, using a pairwise Wilcoxon rank sum test (Holm-corrected), with adjusted p-value threshold <0.05.

We next examined mutation at 70-84 days post infection within previously characterized immunodominant epitopes restricted by common MHC alleles. In all cohorts, both RhCMV/SIV vaccinated and unvaccinated RMs, we detect viral mutation consistent with CD8^+^ T cell immune selection (**Figure 4**). These data suggest once infection is established, *de novo* conventional CD8^+^ T cell responses elicited by the challenge strain provide strong selective pressure in RhCMV/SIV vaccinated RMs, similar to unvaccinated RMs.

## Discussion

RhCMV-based vectors can provide unparalleled control against pathogenic SIVmac239 challenge; however, this protection is observed in just over half of RMs. RhCMV-vectored vaccine protection requires induction of MHC-E restricted CD8+ T cell responses and is associated with the durable presence of a vaccination induced innate immune signature, but factors contributing to the lack of protection in vaccinated animals are not fully elucidated. While viral mutation to evade CD8^+^ T cell responses is a well-documented feature of HIV/SIV and can impact the efficacy of other T cell vaccines, our data demonstrate that viral escape does not explain vaccine failure in the subset of 68-1 RhCMV-vaccinated RMs that were not protected (25). Thus, it is important for future studies to evaluate additional factors that could contribute to these outcomes, such as host genetics or variability in the immune response. Further, while previous studies demonstrate that unconventionally restricted CD8^+^ T cell responses are essential for RhCMV-elicited protection, these responses are not a major driver of intra-host viral evolution in unprotected RMs. Instead, chronic phase viral evolution in unprotected RMs was similar to unvaccinated RMs. Mutations to improve replicative fitness and enable escape from conventional MHC-Ia responses are major drivers of sequence evolution, consistent with published data (26–28). Thus, while unconventionally restricted CD8^+^ T cells play a critical role in the first 7-14 days of infection, if viral control is not established during this window the impact of these responses on viral evolution is limited.

These data are consistent with a very early viral intercept by vaccine-elicited MHC-E-restricted CD8^+^ T cell responses that precedes the post-infection diversification of viral sequence that is substrate of mutational escape from conventional (MHC-Ia-restricted) late-arriving CD8^+^ T cell responses. Since viral sequences are largely identical to the challenge stock at this early timepoint, protection vs. non-protection is more likely related to the functional capacity of the SIV-specific T cells in the early sites of viral infection, a conclusion supported by the correlation of efficacy with the level of activity of the effector-functional promoting cytokine IL-15 (12). The immune function underlying efficacy seems more likely to be related to suppression of viral spread by a cytokine field effect rather than cytolytic activity given both the slow extinction of SIV-infected cells in protected RMs, consistent with gradual decay of an initially infected population rather than rapid active clearance, and as shown here, the absence of escape mutations selected by MHC-E-restricted CD8^+^ T cell responses in non-protected RMs. These observations reinforce the stark immunologic differences between conventional CD8^+^ T cell-mediated elite control and the SIV replication arrest effected by 68-1 RhCMV/SIV vaccine-elicited MHC-E-restricted CD8^+^ T cell responses.

## Data availability statement

The datasets presented in this study can be found in online repositories. Raw sequence data are available in the NIH Short Read Database (SRA) under BioProject PRJNA640430.

## Ethics statement

The animal study was approved by Oregon National Primate Research Center Institutional Animal Care and Use Committee. The study was conducted in accordance with the local legislation and institutional requirements.

## Author contributions

BB: Conceptualization, Data curation, Formal analysis, Methodology, Software, Visualization, Writing – original draft, Writing – review & editing. JS: Data curation, Formal analysis, Methodology, Validation, Writing – review & editing. GM: Formal analysis, Writing – review & editing. JR: Methodology, Project administration, Validation, Writing – review & editing. RP: Methodology, Writing – review & editing. KB: Methodology, Validation, Writing – review & editing. CH: Data curation, Methodology, Project administration, Writing – review & editing. RG: Data curation, Methodology, Project administration, Writing – review & editing. JF: Data curation, Methodology, Project administration, Writing – review & editing. DM: Data curation, Methodology, Project administration, Writing – review & editing. JL: Investigation, Methodology, Validation, Writing – review & editing. JS: Writing – review & editing. SH: Data curation, Investigation, Methodology, Project administration, Supervision, Writing – review & editing. LP: Conceptualization, Funding acquisition, Investigation, Supervision, Writing – original draft.

## Supplementary material

Supplementary Figure 1 Estimation of probability of observing more than zero dominant mutations based upon the sample size of this study. Estimations of the probability of observing a subject with more than zero mutations from a Bayesian binomial model in each cohort, sampled from their posterior probability distribution. Random chance (0.5 probability) is denoted in a vertical dashed line. The credible interval (95% HPDI) in the 68-1 vaccinated cohort is lower than random chance.

Supplementary Figure 2 Plasma viral load data. The graph displays the plasma viral load (PVL) data for each RM used in this study. Data are colored based on vaccine cohort. All RMs established productive SIVmac239 infection and there were no significant differences in chronic phase viral loads between groups. The black arrows denote the two timepoints at which viral sequencing was performed.

Supplementary Table 1 Plasma viral load data.

Supplementary Table 2 Summary of RhCMV vaccine vectors.

Supplementary Table 3 Table of SIVmac239 nucleotide mutation data.

Supplementary Table 4 Table of T cell epitopes used in this study.
