# Supplementary figures and images for: Viral escape mutations do not account for non-protection from SIVmac239 challenge in RhCMV/SIV vaccinated rhesus macaques
Source: Front Immunol. 2024 Aug 7;15:1444621. doi: 10.3389/fimmu.2024.1444621 (PMC11336698; doi:10.3389/fimmu.2024.1444621)

Estimates of Probability of Observing A  
Subject with >0 Non-synonymous Mutations.

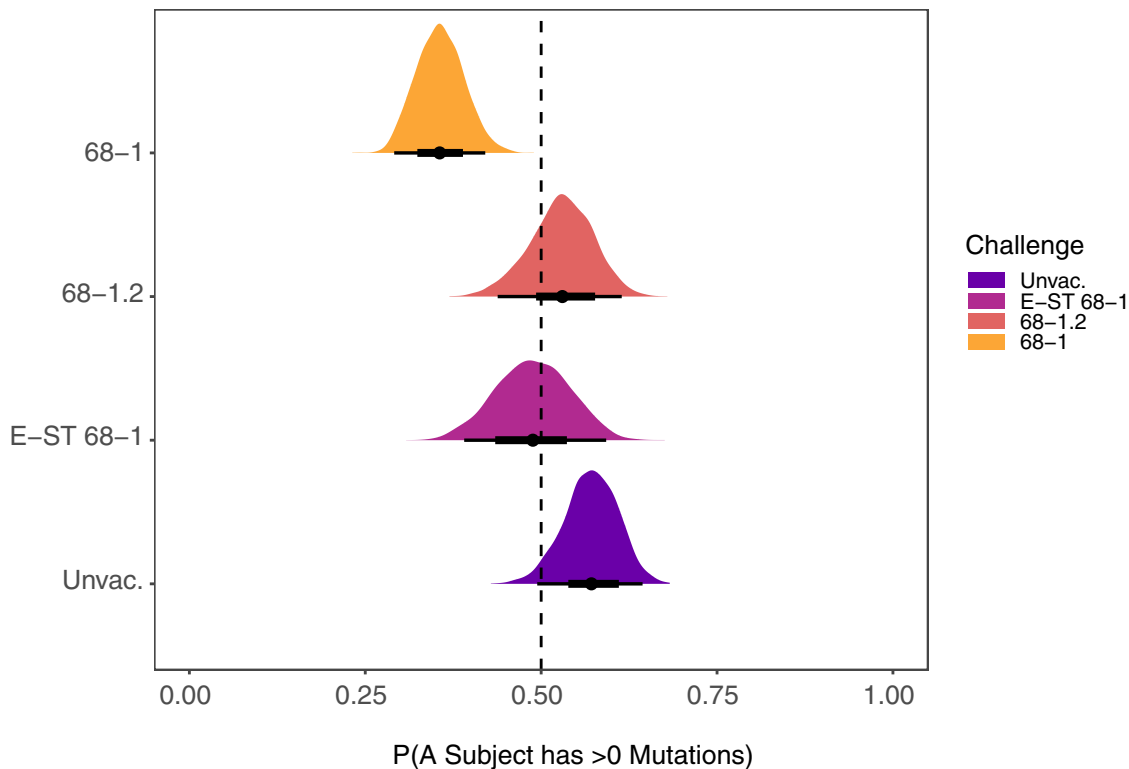

Supplement: Supplementary Figure 1 — Estimation of probability of observing more than zero dominant mutations based upon the sample size of this study. Estimations of the probability of observing a subject with more than zero mutations from a Bayesian binomial model in each cohort, sampled from their posterior probability distribution. Random chance (0.5 probability) is denoted in a vertical dashed line. The credible interval (95% HPDI) in the 68-1 vaccinated cohort is lower than random chance. [file Image_1.pdf]

# Plasma Viral Loads

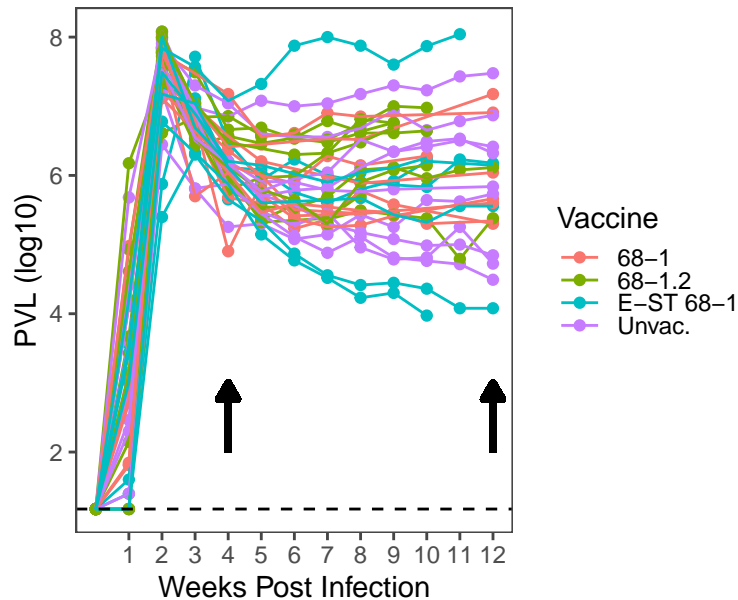

Supplement: Supplementary Figure 2 — Plasma viral load data. The graph displays the plasma viral load (PVL) data for each RM used in this study. Data are colored based on vaccine cohort. All RMs established productive SIVmac239 infection and there were no significant differences in chronic phase viral loads between groups. The black arrows denote the two timepoints at which viral sequencing was performed. [file Image_2.pdf]
